# Perceived Correspondence of Health Effects as a New Determinant Influencing Purchase Intention for Functional Food

**DOI:** 10.3390/nu11040740

**Published:** 2019-03-29

**Authors:** Ágoston Temesi, Ágnes Bacsó, Klaus G. Grunert, Zoltán Lakner

**Affiliations:** 1 Department of Food Economics, Faculty of Food Science, Szent István University, Villányi str. 29-43, 1118 Budapest, Hungary; agi.bacso@gmail.com (Á.B.); lakner.zoltan@etk.szie.hu (Z.L.); 2 MAPP Centre, Department of Management, Aarhus BSS, Aarhus University, Fuglesangsalle 4, DK-8210 Aarhus V, Denmark; klg@mgmt.au.dk

**Keywords:** consumer research, carrier foods, functional ingredients, loglinear analysis

## Abstract

This study has revealed the role of a new factor, perceived correspondence of health effects, in consumer acceptance of functional foods. Using a web survey of 1016 people, we hypothesized and verified the following: when an ingredient does not occur naturally in the carrier but the consumer assigns the same health effect to it as to the carrier, the product’s acceptance will be more positive than it would be if an identical health effect was not associated with the carrier and the functional ingredient. Factors influencing consumer acceptance were examined via binary logistic regression models. According to the results, if a functional food developer fortifies the carrier with an ingredient that does not occur naturally in the carrier, the product can expect higher acceptance if the health effects perceived by consumers are properly matched. In general, it has been found that expected taste and awareness of the product were decisive in all demographic and income groups, whereas perceived correspondence of health effects had a lesser, but still positive influence on acceptance.

## 1. Introduction

Two decades ago Childs [1] claimed that the success of functional foods depends on their consumer acceptance. In spite of comprehensive research on consumer attitudes to functional foods, a decade later Siró et al. [2] (p. 456) still claimed that “the development and commerce of these products is rather complex, expensive and risky, as special requirements should be answered”. Nowadays food fortification is still a significant trend all around the world, which is important for developing countries and for vulnerable consumer groups as well e.g., [3,4,5]. This increases the need for revealing new functional food product development opportunities that keep consumer behavior in mind.

It quickly became obvious in former research that one of the most important aspects of consumer acceptance is taste or expected taste of the product [6,7,8,9,10]. In fact, several studies have concluded that the consumer is not likely to compromise on the product’s taste in exchange for its beneficial health effects [11,12,13,14,15,16]. As Lalor et al. [15] (p. 57) express: “participants were not prepared to purchase foodstuffs if they did not “taste good”, irrespective of health or any other issue”. Although Urala and Lähteenmäki [17] remark that some types of food might have such a strong effect on health that the consumer may renounce their requirements regarding taste, Verbeke’s [18] cross-sectional research confirmed the views of the majority of the scholars, namely that the customer group that accepts an unpleasant taste of a functional food in exchange for its health-promoting effects is rather limited. Verbeke [18] (pp. 130–131) states: “hoping for consumer willingness to compromise on the taste of functional foods for health is highly speculative, risky, and deemed to yield a niche market strategy”.

Several studies have examined how perception of the healthiness status of a carrier influences the acceptance of the functional food. Most of the scholars conclude that consumers more readily accept functional food based on carriers considered healthy [19,20,21,22,23,24,25]. Only Bech-Larsen and Grunert [26] argue with this opinion. In their research, consumers thought fortification of foods considered unhealthy was more reasonable than that of healthy products.

As for the choice of functional ingredient, studies have shown that consumers appreciate if the ingredient used in the enrichment can also be found naturally in the carrier food (e.g., adding calcium to milk, or adding fiber to rye-bread) [7,21,27]. Finally, familiarity with a given functional ingredient significantly influences the acceptance of the food [27]. Urala and Lähteenmäki [8], Messina et al. [28] as well as Krutulyte et al. [29] do not separate familiarity with the new ingredient from familiarity with the whole food, and emphasize the importance of familiarity with the specific functional food. 

Based on this earlier work, Krutulyte et al. [29] developed a conceptual framework for the acceptance of functional foods where the perceived fit of carrier and ingredient is a major determinant of purchase intention, together with health concern and attitude to functional foods. The connection between attitude to fortification and purchase intention has been confirmed recently by e.g., Jahn et al. [30] and Bromage et al. [3], while the connection of perceived fit and purchase intention has been confirmed by Lu [31] and Bruschi et al. [25]. This perceived fit in turn is affected by the expected taste of the functional food, the health image of the carrier, the natural presence of the selected ingredient in the carrier, and finally, familiarity with the product/ingredient. 

## 2. Conceptual Model and Aim of Research

The purpose of the present research was to investigate a new determinant that can influence consumers’ intention to buy functional foods, thus extending the elements of the conceptual model of Krutulyte et al. [29]. The new determinant, called “perceived correspondence of health effects”, is an extension of previous research on perceived fit of ingredient and carrier. Even though an ingredient does not naturally occur in the carrier, the health effect of the two (ingredient and carrier) can be perceived as identical. According to our hypothesis, consumers prefer ingredient/carrier combinations where the ingredient added to the carrier possesses the same perceived health effect as the carrier itself, compared to those where the perceived health effects of carrier and ingredient are different. The research of Krutulyte et al. [29] indicated a connection between purchase intention and perceived fit of carrier–ingredient combination, suggesting that a “correspondence of health effects” would influence the purchase intention for functional foods indirectly.

**Figure 1:**
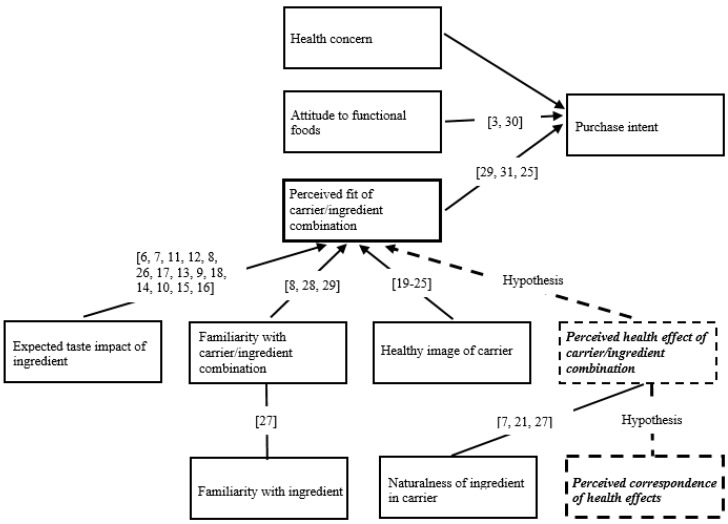
Determinants (confirmed and hypothesized) influencing purchase intent for functional foods.

The hypotheses of the research are visualized in Figure 1. 

Figure 1 shows our conceptual framework. It is based on the original work by Krutulyte et al. [29] extended with our hypothesis and indicating the references which confirm the effects of the different influencing factors. We hypothesize that the perceived match of the health effects of carrier and ingredient, together with the naturalness of the ingredient in the carrier, affects the perceived overall health effect of carrier/ingredient combination. This is based on the argument that an increased health effect of the product can be obtained in two ways: by increasing the amount of the healthy ingredient naturally contained in the product (enrichment), or by adding a new component which has the same perceived health effect as the product (fortification). Our main hypothesis is that the perceived correspondence of health effects in a carrier/ingredient combination directly increases the perceived fit of carrier/ingredient combination. 

The aim of research has been the empirical testing of this conceptual model and notably our main hypothesis. The ultimate aim of research has been to empirically test a new approach to functional products.

If this hypothesis was supported, it would expand the options of product developers, since not only enriching the product with ingredients which naturally occur in the carrier will be appropriate, but also all ingredients to which consumers assign the same health effect as to the carrier. 

## 3. Materials and Methods 

A non-representative consumer survey was carried out to investigate the research hypothesis. Respondents were asked to complete a 10-min online questionnaire in the first two weeks of October 2015. We shared the Hungarian language survey in social media using a snowball sampling method started with Hungarians. 

Respondents were informed that the aim of the questionnaire was to measure factors that might influence their purchase intent for functional foods. All respondents filled out anonymous questionnaires and they could quit without submitting it at any point of the questionnaire. Respondents filled out the questionnaire as volunteers and did not receive any incentives.

### 3.1. Characteristics of the Sample

1016 people provided valid answers. The composition of the sample is shown in Table 1. 

Participants in the online survey were mostly women, between the ages of 18 and 29, and with relatively high education (secondary or tertiary); facts that can be attributed to the methodology of the survey and to these groups’ interest in the issue as we have used the social media to recruit respondents. Recent findings however show that functional components are more important for women and better educated people [32].

### 3.2. Composition of the Questionnaire 

Five carriers have been selected for the product combinations (100% pure orange juice, muesli bar from oat-flakes, natural yogurt, dark chocolate with at least 70% cocoa content, rye-bread), which were combined with 11 types of functional ingredients (vitamins A, E, C, and D, calcium, magnesium, probiotics, linseed, oat-flakes, ginseng, caffeine). Of the 55 possible carrier/ingredient combinations, only those were retained where the ingredient does not naturally occur in the carrier or occurs only in traces according to nutritional experts we have consulted. This resulted in 28 remaining combinations as shown in Table 2. 

The questionnaire contained measures corresponding to the constructs in Figure 1. The questions of the questionnaire were developed by the authors.

The perceived healthiness of the carrier food was measured on a semantic differential scale from 1 (very unhealthy) to 5 (very healthy). In a similar way, the expected taste of product combinations was measured on a semantic differential scale from 1 (having a very bad taste) to 5 (very tasty). Familiarity was measured by asking whether respondents had seen the product combination before and, if yes, if they had tasted it. 

In order to measure the perceived health effects of the 5 carriers and 11 functional ingredients, five statements about possible health effects were formulated after consultation with nutritional experts. From these respondents could choose the ones they associated with the ingredients and carriers. The statements were the following: “strengthens the immune system and adds to general well-being”, “helps digestion and gastrointestinal functions”, “increases mental performance”, “supports the formation of healthy bone weight and the health of bones”, and finally “can reduce the risk of heart and other cardiovascular diseases, reduces the cholesterol level of the blood”. Respondents could also choose “I do not know” answer. Based on these data the variable “perceived correspondence of health effects” was formed. It had a score of 1 when the respondent marked at least one common health effect for both the carrier of the combination and the functional ingredient and a score of 0 otherwise.

In the next part of the questionnaire, respondents were asked to evaluate the 28 product combinations on the extent to which the carrier and the ingredient fit. Again, a scale from 1 (does not think the carrier and ingredient fit) to 5 (believe that the carrier and ingredient fit appropriately) was applied. 

Before the beginning of the web-based consumer survey, the questionnaire was pre-tested in individual interviews to check the wording and clarity of the questions.

### 3.3. Data Analysis

Preliminary analyses of the scales used for the determinants indicated that they have a skewed distribution, with answers concentrated at the higher or lower scale values. For further analysis, the scale values were therefore transformed to binary variables. For the perceived healthiness of the carrier food, the results were transformed such that values of 4 and 5 were converted into a value of 1, indicating a positive consumer judgement on healthiness, while smaller values were converted into 0, indicating a low level of perceived healthiness. Likewise, for expected taste, scores 4 and 5, showing that respondents believe the product combination has a good taste, were converted into 1, while lower values were converted into 0. Also, for ratings of perceived fit between carrier product and ingredient, values 4 and 5 (indicating good fit) were transformed to 1, whereas lower values (indicating little perceived fit) were converted to 0.

Familiarity was coded based on previous exposure to the combination. Having seen but not tasted the product and having seen and tasted the product were recoded into 1 and named as awareness, and having neither seen nor tasted the product was recoded into 0.

A new variable “correspondence of health effects” was formed which was assigned the value 1 if the respondent marked at least one common health effect for both the carrier of the combination and the functional ingredient, and 0 otherwise.

A pooled set of data was formed. The original table recorded 1016 rows of observations. The analysed four determinants and the purchase intention for 28 product combinations as well as the demographic variables of respondents were listed. We “unstacked” the data into stacked form. In this way, all of the data for a determinant (e.g., perceived correspondence of health effects) have been grouped together in a single column and finally, we had 28448 (1016 × 28) records. This data structure allowed us to analyse the effect of different factors.

Determinants of perceived fit between carrier product and ingredient were examined by binary logistic regression models. Because of the large number of predictors, and to reduce the distorting effect caused by the relations among them, binary logistic regression with stepwise backward elimination method was applied. The removal testing has been based on the probability of the likelihood-ratio statistic (*p* ≤ 0.05), based on the maximum partial likelihood estimates. The analysis was carried out individually for all 28 carrier/ingredient combination and for the pooled data. After assessing the influencing factors for the whole sample, we repeated the analysis for different demographic groups. The effects of determinants on acceptance of product combinations in different socio-economic groups were also estimated based on the pooled data. After the linear regression analysis, we simply counted and summarized the number of significant differences between various factors. All variables were also checked for relationships with demographic characteristics. All statistical analyses were done at the 5% significance level (*p* ≤ 0.05).

## 4. Results and Discussion

### 4.1. Role of Determinants in Perceived Carrier/Ingredient Fit

The main aim of our research was to test whether correspondence of health effects has an impact on perceived fit between carrier and ingredient, and to compare the size of the effect with other determinants that in previous research have been shown to have an effect on perceived fit. Results of the binary logistic regressions are in Table 3.

The influence of the new determinant, perceived correspondence of health effects, on consumer perception of carrier/ingredient fit is different both by product group and by each individual combination. The strongest effect can be found in the following combinations, in descending order: muesli-bar–linseed, rye-bread–oat-flakes, yogurt–oat-flakes, and rye-bread–linseed. Consumer opinion least depends on perceived correspondence of health effects in the case of the combinations of dark chocolate (independently of the applied bioactive component) and of products containing ginseng.

The role of correspondence of health effects could not be shown in any of the products fortified with ginseng, which is probably due to the fact that this herb is not well- known in Hungary, so its effects also remain hidden from the public. As for dark chocolate, Balasubramanian and Cole [19] provide an explanation. Their research found that for products belonging to the category “fun foods”, nutritional information is less important than the need for a good taste, so the researchers do not recommend their use as carriers. This is in line with the results of the present research.

The results support the importance of the health image of the carrier food in consumer perception of functional foods, a determinant that has been researched by many scholars. An effect is found in several combinations of all five food groups; at the same time, independently of the combinations, a significant link has been demonstrated between considering a carrier food healthy and attitudes towards its carrier/ingredient combination. Based on our results, we share the opinion of other scholars [19,20,21,22,23,24] that the perception and market success of a functional food is enhanced if the fortified carrier is well-known for being healthy.

Expected taste turns out to be the most important determinant for all examined combinations and also in general. The results are in accordance with the findings of previous research e.g., [6,7,8,9,10,11,12,13,14,18].

Among the carriers, the role of taste is most prominent in the examined combinations of yogurt, where the chance that the consumer perceives a fit between carrier and ingredient is 4.3 times higher if the resulting taste is expected to be pleasant. This value is significantly higher than the one for the examined combinations of orange juice, where an expected good taste results in a 3-fold increase in perceived fit. In the combinations of the muesli bar, taste plays the most important role, too; nonetheless, awareness of the combinations was also very important, and for some combinations (linseed, magnesium, probiotics), the perceived correspondence of health effects also had a high chance quotient. 

Awareness is a determining factor mostly in the case of product concepts containing yogurt, rye-bread, and oat-flakes, or linseed. The results concerning awareness of the combinations confirm the findings of Grunert et al. [27], according to which consumer attitude (in the researched combinations and in general as well) is more favourable if the respondent has already met the carrier–ingredient combination earlier. This underlines the importance of marketing communication or any other activity that focuses on introducing the product to consumers or informing the public about the product.

### 4.2. Role of Determinants in Perceived Carrier/Ingredient Fit in Case of Socio-Demographic Groups

The factors influencing the perception of the 28 carrier/ingredient combinations have been examined according to the respondents’ gender, age group, residence, schooling and income perception. Table 4 shows how many of the 28 examined product combinations were significantly influenced by the 4 predictors within the different demographic subgroups. 

Results show that in almost all of the examined 28 product combinations, expected taste was significantly related to perceived fit in all respondent groups. It is also obvious that for men, for those from villages, for people with an education below secondary level, and for people with below average income, this is the only factor that influences their perceived fit of a carrier/ingredient combination. In contrast, women are influenced by more factors when judging the fit of a product concept. 

For respondents between the ages of 18–29 and for those who live in the capital, awareness of the product is important in several cases. Beside awareness of the product combination, the health image of the carrier is also of great importance for those older than 30, for people who live in a town/city, for those who have tertiary education, and for women.

Perceived correspondence of health effects influenced perceived fit of a carrier/ingredient combination mostly among women, for those with secondary education, and for people with an average income. With the exception of male respondents, perceived correspondence of health effects had a favourable influence on perceived fit for at least some of the 28 product combinations.

Table 5 shows the same analysis for the data pooled across all carrier/ingredient combinations.

The results indicate that the examined determinants have a positive influence on the attitudes towards a carrier–ingredient combination in all socio-demographic and perceived income groups. In general, the expected taste of the combination had a great importance. On average, expected good taste resulted in a 3.6 times bigger chance of perceived fit, with some cases reaching a 5-fold increase. This factor was especially important for those with primary education, people with below average income, those older than 30, and for people from the capital. Another aspect of great importance was awareness of the combination for all respondent groups, but especially for women, young people and for those from the capital. Health image of the carrier, verified in previous research, and perceived correspondence of health effects, verified in the present study, both positively influence perceived fit of carrier/ingredient combination in each respondent group, although to a lesser extent than the two other determining factors. Specifically, perceived correspondence of health effects led to a 30% bigger chance of perceived fit.

### 4.3. Managerial Implications 

“Ensure healthy lives and promote well-being for all at all ages” is considered as a priority, being part of the Sustainable Development Goals (Agenda 2030) of the United Nations [33]. The majority of leading food producers are committed to enhancing the portfolio of functional products. 

Due to these, it is a very important question, how to motivate the consumers to choose the alternatives that are beneficial for their health. The understanding of interrelationships between functional ingredients, carriers, and consumers’ preference system can be an important aid in the phase of conceptualisation, development, and promotion of novel food products. These pieces of knowledge can be important in decreasing the failure rate of the launching of new products and could contribute to increasing the efficiency of the utilisation of R+D funds.

### 4.4. Limitations

As a limitation of the research, we have to add that it has been carried out in Hungary using a web survey for the non-representative, convenient sampling of Hungarian respondents, which might cause the sample to be biased. This should be kept in mind when considering the results. We have tested our hypothesis for only 28 product combinations. International researches with increased number of possible carrier ingredient combinations could improve the generalizability of our findings. More types of demographic variables and representative sampling could help finding the target group where perceived correspondence of health effects has the strongest effect on purchase intention. 

## 5. Conclusions

We have examined the effect of different determinants on the perceived fit of 28 carrier/ingredient combinations created from 5 carriers and 11 ingredients. In the case of all individual products and also in general, it was found that expected taste had the greatest influence on the perceived fit of a carrier–ingredient combination. At the same time, awareness of the combination, the health image of the carrier and perceived correspondence of health effects also had an influence. Nonetheless, the role of these determinants is different for each carrier food. The largest variances between the various combinations were found in connection with perceived correspondence of health effects. This factor has the highest influence on the perceived fit of muesli-, rye-bread-, and yogurt-based product combinations, while in the case of dark chocolate no such effect was found. 

As a summary, it can be stated that for numerous products, enhancing an existing and well-known health effect of the carrier increases positive perceived fit even if the ingredient is not originally present in the carrier. 

In the case of almost all product combinations in all respondent groups, a substantial influence of the expected taste was found. Perceived correspondence of health effects positively influenced the perceived fit of the carrier/ingredient combinations among women, among those with secondary education and among people with average income.

When generalising over the individual acceptance of products, it was found that beside taste, awareness of the product (that is whether the respondent has heard of the given product or not) is also of great importance when forming an opinion about it. At the same time, perceived correspondence of health effects was proven to be a positive influencing factor in all respondent groups.

It can be stated that in addition to the influencing factors researched in previous studies, perceived correspondence of health effects also plays an important role in whether consumers perceive a good fit between a carrier product and a functional ingredient. Nonetheless, the effect of individual determinants differ significantly in each food category and combination.

## Figures and Tables

**Table 1:** Socio-demographic characteristics of the sample.

Socio-Demographic Characteristics	%
*Gender*	
female	88.7
male	11.3
*Age group*	
18–29	51.4
30–44	20.7
45 or older	27.9
*Residence*	
village	15.7
town/city	43.1
capital	41.1
*Education*	
primary	6.3
secondary	49.5
tertiary	44.2
*Perceived income*	
below average	17.2
average	67.1
above average	15.7

**Table 2:** Carrier foods and the ingredients combined with them.

Carrier Foods	100% Pure Orange Juice	Muesli Bar from Oat-Flakes	Natural Yogurt	Chocolate with at Least 70% Cocoa Content	Rye-Bread
**Functional ingredients**	Vitamin A Vitamin D Vitamin E Calcium Ginseng	Vitamin C Vitamin D Magnesium Probiotics Linseed	Vitamin E Oat-flakes Linseed Ginseng	Vitamin C	Vitamin C
				Vitamin D	Vitamin D
				Calcium	Calcium
				Magnesium	Oat-flakes
				Probiotics	Linseed
				Ginseng	Probiotics
				Caffeine	Ginseng

**Table 3:** The role of the influencing factors in the fit perception of 28 functional food carrier/ingredient combinations (*n* = 1016, 28 answers per respondent).

	Perceived Correspondence of Health Effects (expB)	Health Image of Carrier (expB)	Expected Taste of Combination (expB)	Awareness of the Combination (expB)
Orange juice + vitamin A	1.329 *	2.200 **	2.928 **	1.800 **
Orange juice + vitamin E	1.449 **	2.007 **	2.723 **	1.482 *
Orange juice + ginseng			3.493 **	
Orange juice + calcium		2.273 **	2.728 **	2.138 **
Orange juice + vitamin D		1.979 **	2.626 **	2.317 **
Muesli bar + probiotics	1.668 **		2.917 **	1.480 *
Muesli bar + linseed	2.160 **		5.546 **	1.604 **
Muesli bar + vitamin C	1.422 *		3.706 **	1.691 **
Muesli bar + magnesium	1.700 **	1.379 *	3.360 **	1.417 *
Muesli bar + vitamin D		1.361 *	3.967 *	
Yogurt + linseed	1.625 **		5.033 **	2.161 **
Yogurt + oat-flakes	1.887 **	1.740 *	5.040 **	4.589 **
Yogurt + vitamin E	1.606 **	2.480 **	2.810 **	1.620 **
Yogurt + ginseng		1.807 *	4.328 **	2.632 **
Dark chocolate + caffeine			3.565 **	2.642 **
Dark chocolate + ginseng		1.586 **	3.764 **	
Dark chocolate + calcium		1.565 **	2.770 **	1.428 *
Dark chocolate + magnesium		1.305 *	2.980 **	
Dark chocolate + vitamin C		1.458 **	3.608 **	2.189 **
Dark chocolate + probiotics		1.483 **	3.375 **	
Dark chocolate + vitamin D			3.912 **	2.083 *
Rye-bread + linseed	1.880 **	1.531 *	4.859 **	2.734 **
Rye-bread + oat-flakes	2.041 **	1.527 *	4.439 **	2.924 **
Rye-bread + calcium	1.550 *	1.604 **	2.866 **	2.567 **
Rye-bread + vitamin C		1.537 *	3.728 **	2.412 **
Rye-bread + probiotics	1.475 *		3.634 **	
Rye-bread + ginseng		1.577 **	5.199 **	2.480 **
Rye-bread + vitamin D	1.440 *	1.613 **	3.179 **	2.021 *
All carrier/ingredient combinations	1.330 **	1.425 **	3.606 **	2.556 **

* *p* ≤ 0.05; ** *p* ≤ 0.01.

**Table 4:** The number of functional food combinations (out of 28) where perception of carrier/ingredient fit was significantly affected by predictors within different demographic groups.

	Perceived Correspondence of Health Effects	Health Image of the Carrier	Expected Taste of the Combination	Awareness of the Combination
male	0	2	26	6
female	14	17	28	22
ages 18–29	9	5	28	15
ages 30–44	5	15	27	11
45 or older	9	13	28	8
village	4	8	25	6
town/city	7	15	28	16
capital	6	7	28	15
primary education	2	5	25	2
secondary education	13	7	28	22
tertiary education	6	10	28	10
income below average	1	3	28	8
average income	11	13	28	17
income above average	3	11	26	9

* *p* ≤ 0.05; ** *p* ≤ 0.01.

**Table 5:** The influence of individual factors on the perceived fit of carrier/ingredient combinations in different demographic groups.

	Perceived Correspondence of Health Effects (expB)	Health Image of the Carrier (expB)	Expected Taste of the Combination (expB)	Awareness of the Combination (expB)
male	1.23 *	1.29 **	3.59 **	2.15 **
female	1.33 **	1.43 **	3.63 **	2.63 **
Age 18–29	1.29 **	1.19 **	3.16 **	2.96 **
Age 30–44	1.34 **	1.87 **	4.12 **	2.59 **
45 or older	1.36 **	1.88 **	4.11 **	2.03 **
village	1.27 **	1.95 **	2.96 **	2.18 **
Town/city	1.41 **	1.56 **	3.40 **	2.50 **
Capital	1.26**	1.17 **	4.14 **	2.84 **
Primary education	1.68 **	2.32 **	5.18 **	1.62 **
Secondary education	1.34 **	1.41 **	3.18 **	2.66 **
Tertiary education	1.28 **	1.36 **	3.96 **	2.62 **
Income below average	1.39 **	1.41 **	5.05 **	2.37 **
Average income	1.36 **	1.35 **	3.38 **	2.54 **
Income above average	1.15 *	1.81 **	3.36 **	2.94 **

* *p* ≤ 0.05; ** *p* ≤ 0.01.
